# A Case of Postpartum Preeclampsia With an Unusual Presentation

**DOI:** 10.7759/cureus.96261

**Published:** 2025-11-06

**Authors:** Akiva Eleff, Mendel Shloush, Brian Bugay

**Affiliations:** 1 Emergency Medicine, Ohio University Heritage College of Osteopathic Medicine, Cleveland, USA; 2 Orthopedic Surgery, Florida International University (FIU) Herbert Wertheim College of Medicine, Miami, USA

**Keywords:** atypical preeclampsia, bradycardia, postpartum complications, postpartum hypertension, respiratory distress, severe features, sinus bradycardia, unusual presentation, women health

## Abstract

Preeclampsia is a hypertensive disorder of pregnancy that usually presents with elevated blood pressure. It may also occur in the postpartum period with atypical features, which can complicate timely recognition and adequate treatment. We report a case of a 33-year-old female patient who presented five days postpartum with chest tightness and shortness of breath. Initial findings were atypical for preeclampsia. She was severely bradycardic and normotensive, though hypoxic and tachypneic. Workup excluded postpartum cardiomyopathy and pulmonary embolism (PE), but revealed small bilateral pleural effusions. Within three hours, her blood pressure rose to 160/90 mmHg. Given the rapid onset of severe hypertension combined with laboratory evidence of multisystem organ involvement, including mildly elevated liver enzymes, proteinuria, and pleural effusions, she was diagnosed with preeclampsia with severe features. Management included oxygen and antihypertensive therapy with hydralazine. She was safely discharged two days later. This case is notable because the patient presented with bradycardia and normotension, which are uncommon findings in preeclampsia. The subsequent rise in blood pressure, combined with pleural effusions, supported the diagnosis once other cardiopulmonary causes were excluded. Careful evaluation, timely treatment, and appropriate monitoring led to a favorable outcome. This highlights the importance of considering preeclampsia in postpartum patients with atypical presentations.

## Introduction

Approximately 10% of pregnancies are affected by hypertensive disorders [1], which are broadly defined by the International Society for the Study of Hypertension in Pregnancy (ISSHP) as the development of high blood pressure (≥140/90 mmHg) after 20 weeks of gestation [2]. This group of conditions includes gestational hypertension, chronic hypertension, and preeclampsia, whether occurring independently or on top of chronic hypertension.

These disorders can pose serious health risks to both the mother and fetus, not only during pregnancy but also well beyond delivery. For mothers, long-term risks include a two- to four-fold increase in chronic hypertension, twice the likelihood of cardiovascular-related death and major cardiac events, and a 1.5-fold elevated risk of stroke [3]. 

Preeclampsia is a multisystem disorder of pregnancy, classically defined as new-onset hypertension (≥140/90 mmHg) and either proteinuria or evidence of maternal organ dysfunction after 20 weeks of gestation. Typical presentations include hypertension, proteinuria, and symptoms or laboratory evidence of renal, hepatic, neurologic, or hematologic involvement. Severe features include marked hypertension, altered mental status, visual disturbances, severe headache, right upper quadrant pain, and significant transaminase elevations [4,5].

Atypical presentations are well documented. Preeclampsia can rarely develop before 20 weeks of gestation, most often in cases of molar pregnancy or fetal hydrops, and should be considered when classic features are present in these contexts [6,7]. Postpartum preeclampsia may occur after 48 hours and up to several weeks following delivery, with similar clinical and laboratory findings as antepartum disease [6,8].

Some patients present without hypertension, instead manifesting with end-organ dysfunction such as altered mental status (eclampsia, encephalopathy) or severe elevations in liver transaminases (aspartate aminotransferase (AST)/alanine aminotransferase (ALT) >1000 U/L), which may mimic acute hepatic injury. These cases require a high index of suspicion, as delivery is the definitive treatment to prevent maternal morbidity and mortality [4,9,10]. The American Association for the Study of Liver Diseases highlights that hepatic involvement can be profound, and the diagnosis should be considered even in normotensive patients with severe transaminitis and neurologic symptoms [9].

Similarly, autonomic manifestations such as bradycardia may occur in atypical or severe cases and can obscure the clinical picture, particularly when blood pressure remains within normal limits at presentation. These uncommon findings, as seen in our patient, highlight the diagnostic complexity of postpartum preeclampsia and underscore the importance of considering the disorder even when classic hypertensive features are initially absent.

We present a case of a patient five days postpartum who initially suffered from severe bradycardia and normotension. Although rare cases of postpartum bradycardia have been associated with preeclampsia [11,12], the presence of initial normotension with this severe bradycardia makes this presentation particularly noteworthy. This case adds to the existing literature by highlighting that preeclampsia with severe features can evolve rapidly, even when initial vital signs, such as blood pressure, appear reassuring. It therefore emphasizes the need for vigilance in identifying unusual clinical signs in postpartum patients, particularly those with relevant medical histories.

## Case presentation

A 33-year-old female patient, five days postpartum following a cesarean section delivery, presented to the emergency department with complaints of chest tightness and shortness of breath. Her medical history included dilation and curettage, a recent tubal ligation, and a recent diagnosis of iron deficiency anemia.

Upon initial examination, the patient's presentation was atypical for severe preeclampsia, marked by significant respiratory distress but an unremarkable blood pressure reading. She was tachypneic, with a respiratory rate in the upper 30s. Initial oxygen saturation was 90% on room air, which required 4 L of oxygen per minute via nasal cannula to normalize her blood oxygen levels. Auscultation revealed bilateral rales. Crucially, her initial blood pressure was normotensive at 130/70 mmHg, and her heart rate was profoundly bradycardic at 36 bpm. There were no signs of edema or neurological deficits.

Given the hypoxia and bradycardia in the postpartum setting, the initial concern was for pulmonary embolism (PE) or postpartum cardiomyopathy. This led to an appropriate clinical workup, which included ordering a chest X-ray, which was unremarkable for any acute cardiopulmonary process. A transthoracic echocardiogram (TTE) was performed with the following findings: the left and right ventricles were normal in size with normal systolic function (left ventricle ejection fraction (LVEF): 57±5%), and there were no significant valvular abnormalities. A CT angiogram (CTA) study was performed and revealed small bilateral pleural effusions, but no PE was noted.

Laboratory tests revealed a hemoglobin level of 9.4 g/dL, consistent with iron deficiency anemia. Liver function tests showed mildly elevated ALT (55 U/L) and AST (52 U/L). Kidney function was normal, with creatinine levels within the normal range. Urinalysis revealed proteinuria (+1) and a protein/creatinine ratio of 0.24. High sensitivity troponin T levels were within normal limits at 12 ng/L. The summarized results of the laboratory investigations are presented in Table 1.

**Table 1:** Summary of laboratory investigations Reference ranges may vary slightly by laboratory; female ranges are cited where applicable.

Parameter	Result	Reference Range	Interpretation
Hemoglobin	9.4 g/dL	12.0-16.0 g/dL (female)	Decreased (anemia)
Alanine aminotransferase (ALT)	55 U/L	7-35 U/L	Mildly elevated
Aspartate aminotransferase (AST)	52 U/L	8-33 U/L	Mildly elevated
Creatinine	0.8 mg/dL	0.6-1.1 mg/dL (female)	Within normal limits
Urinalysis: protein	+1	Negative	Mild proteinuria
Urine protein/creatinine ratio	0.24	<0.2	Mildly elevated
Troponin	12 ng/L	<14 ng/L	Within normal limits

Three hours after admission, the patient's blood pressure increased dramatically to 160/90 mmHg. Given this abrupt rise in blood pressure, the worsening symptoms, and the presence of pleural effusions, the diagnosis was rapidly shifted to preeclampsia with severe features.

The clinical decision-making regarding management was guided by the patient’s respiratory status. The obstetrics/gynecology (OB/GYN) on call recommended initiating hydralazine for blood pressure control once the systolic pressure exceeded 150 mmHg or the diastolic pressure exceeded 110 mmHg. Magnesium sulfate administration was advised against due to the patient’s existing significant respiratory distress and tachypnea, as this medication could worsen respiratory function and compromise the patient's airway. Close observation and prudent medical testing in this unique case enabled the patient to have well-controlled medical care, and she was safely discharged two days later.

## Discussion

Preeclampsia is a hypertensive disorder of pregnancy, typically manifesting after 20 weeks of gestation, but it can also occur in the postpartum period. Postpartum preeclampsia is defined as new-onset hypertension and proteinuria occurring after delivery and can lead to severe maternal morbidity and mortality if not appropriately managed. This patient’s case was complicated by an unusual presentation of normotension upon initial presentation and sinus bradycardia, which is rarely associated with preeclampsia [13].

Bradycardia in postpartum preeclampsia is infrequent but has been documented, particularly in the setting of severe forms of the disorder [13]. The initial presentation of profound bradycardia (heart rate of 36 bpm) and acute respiratory distress posed a major diagnostic challenge, leading to an initial differential diagnosis that included primary cardiac pathology. However, a systematic workup effectively excluded primary cardiac etiologies: PE, a concern given the hypoxia and postpartum status, was ruled out by a negative CT angiogram. Crucially, the TTE revealed preserved left ventricular function, allowing us to formally exclude peripartum cardiomyopathy.

The most widely supported mechanism of bradycardia in preeclampsia is autonomic imbalance, specifically an increase in parasympathetic (vagal) tone or a relative withdrawal of sympathetic activity, which can occur in certain phases of preeclampsia, particularly in late-onset or delayed postpartum cases. Heart rate variability studies demonstrate that late-onset preeclampsia is associated with increased baroreceptor reflex sensitivity and higher parasympathetic modulation, which can lead to relative sinus bradycardia compared to healthy pregnancies [14]. This contrasts with early-onset preeclampsia, where sympathetic overactivity predominates [15,16].

In delayed postpartum preeclampsia, case reports and small series suggest that increased vagal tone may be triggered by abrupt hemodynamic shifts, endothelial dysfunction, and changes in circulating vasoactive substances after delivery, resulting in transient bradycardia [14]. Additionally, autonomic dysregulation with fluctuating sympathetic and parasympathetic activity may underlie episodes of bradycardia, especially when baroreceptor reflexes are exaggerated in response to hypertension or volume shifts [14].

In the context of hemolysis, elevated liver enzymes, and low platelets (HELLP) syndrome, bradycardia may precede acute hemodynamic changes. Proposed mechanisms include hepatic injury and systemic inflammation leading to altered autonomic output, as well as direct effects of cytokines and vasoactive mediators on cardiac conduction pathways [5,17]. Cardiac conduction abnormalities and reduced heart rate variability have been observed in women with preeclampsia and HELLP, supporting a link between autonomic dysfunction and bradycardia [18].

A related mechanism that may coexist with this autonomic imbalance is an exaggerated baroreceptor reflex response to rapidly increasing systemic vascular resistance and acute hypertension, which has been proposed in case reports as a cause of transient bradycardia in severe preeclampsia [19,20]. The underlying physiological cascade originates from abnormal placentation and resultant placental insufficiency, which trigger the release of proinflammatory factors and oxidative stress mediators. These, in turn, lead to widespread maternal endothelial dysfunction, intense vasoconstriction, and markedly elevated afterload on the heart [21,22]. This sequence can explain the patient’s rapid clinical deterioration as well as the associated findings of mild proteinuria and elevated liver transaminases observed in this case.

However, despite the absence of classic hypertension at the time of presentation, the rising blood pressure, combined with respiratory distress and abnormal laboratory findings, ultimately led to a diagnosis of preeclampsia with severe features. The treatment of postpartum preeclampsia typically involves blood pressure control, often with antihypertensive medications such as hydralazine [23], as was done in this case. Magnesium sulfate is another common therapy for severe preeclampsia, but it was appropriately withheld due to the patient’s respiratory status [24]. Close observation is essential, and the patient in this case showed favorable outcomes with appropriate medical management. This case emphasizes the importance of maintaining a high index of suspicion for preeclampsia in the postpartum period, even in the absence of classic hypertension. Early identification and timely intervention can prevent complications such as eclampsia, stroke, and organ failure.

## Conclusions

This case underscores the critical importance of careful monitoring and management of postpartum patients for signs of preeclampsia, even in the absence of classic hypertension. The patient's initial presentation, characterized by profound bradycardia and normotension despite severe respiratory distress, highlights that this life-threatening disorder may mimic a primary cardiac event. Early identification and appropriate management, including the exclusion of primary cardiac disease via TTE and CTA and timely treatment of hypertensive surges, are essential to prevent severe complications. For clinicians, this case serves as a vital reminder to maintain a high index of suspicion for evolving preeclampsia when faced with atypical symptoms and nonclassical vital signs in the postpartum period. The patient was safely discharged with follow-up instructions and recommendations for ongoing monitoring of her blood pressure and symptoms.
